# Miniature Surface Plasmon Polariton Amplitude Modulator by Beat Frequency and Polarization Control

**DOI:** 10.1038/srep32098

**Published:** 2016-08-25

**Authors:** Cheng-Wei Chang, Chu-En Lin, Chih-Jen Yu, Ting-Tso Yeh, Ta-Jen Yen

**Affiliations:** 1 Department of Materials Science and Engineering, National Tsing Hua University, 101, Section 2, Kuang Fu Road, Hsinchu 30013, Taiwan; 2 Department of Mechanical Engineering, National Chin-Yi University of Technology, Taichung 41170, Taiwan; 3 Graduate Institute of Electro-Optical Engineering, Chang Gung University, Taoyuan 333, Taiwan; 4 Center for Nanotechnology, Materials Science, and Microsystems, National Tsing Hua University., 101, Section 2 Kuang Fu Road, Hsinchu 30013, Taiwan.

## Abstract

The miniaturization of modulators keeps pace for the compact devices in optical applications. Here, we present a miniature surface plasmon polariton amplitude modulator (SPPAM) by directing and interfering surface plasmon polaritons on a nanofabricated chip. Our results show that this SPPAM enables two kinds of modulations. The first kind of modulation is controlled by encoding angular-frequency difference from a Zeeman laser, with a beat frequency of 1.66 MHz; the second of modulation is validated by periodically varying the polarization states from a polarization generator, with rotation frequencies of 0.5–10 k Hz. In addition, the normalized extinction ratio of our plasmonic structure reaches 100. Such miniaturized beat-frequency and polarization-controlled amplitude modulators open an avenue for the exploration of ultrasensitive nanosensors, nanocircuits, and other integrated nanophotonic devices.

A wave modulator, a demanded device that encodes information into a wave carrier, is a key component in optoelectronic applications because it promises channel miniaturization, broad transmission bandwidth, and high signal-to-noise (S/N) ratio of the output^1^,^2^,^3^. To date, researchers have realized a variety of advanced modulators by employing electro-optic crystals^4^, phase transition materials^5^, Bragg cells^6^,^33^, and liquid crystals^4^. To further enhance the performance of the demanded modulator, a new concept of plasmonic modulators has attracted increasing attention from researchers. A main advantage of plasmonic modulators is their miniaturized sizes that can be even smaller than the diffraction limit because surface plasmon support super-oscillating spatial frequency^7^. Therefore, not only can plasmonic modulators effectively reduce the long-range distance of signal propagation for the phase delay^8^, but they can also provide on-chip solutions such as Si modulators^9^, plasmonic phase modulators^2^, and gap plasmon phase modulators (GPPM)^3^.

A plasmonic modulator can certainly be compact, but it requires a coupler to compensate the mismatched wave momentum between the propagating mode and the surface mode^7^. Therefore, several couplers were recently demonstrated, such as nanoslits^10^,^11^ and nanogratings^12^; yet, these sub-wavelength structures cannot control the propagation direction of surface plasmon polaritons (SPP) under normal incidence, and were found to be sensitive to the polarization states^10^,^11^,^12^. More recently, several types of unidirectional couplers have appeared that guide the SPP waves by breaking the phase symmetry and controlling the polarization states, such as an aperiodic groove^13^, a double slit^14^, a guiding-mode controller^15^,^16^,^18^, a dipole mode rotator^32^, and a Fishbone (FB) structure^17^. Among these unidirectional couplers, the FB structure exhibits further advantages of directing the SPP by the specific polarization states, and of exciting the SPP under the normal incidence^17^.

Herein, we focus on the issue of optical channel miniaturization for the plasmonic amplitude modulation to effectively reduce the optical paths and the power dissipations. The integration of the FB coupler and the continuous wave (CW) lasers is developed for the SPP amplitude modulator (SPPAM). Such a SPPAM enables the modulation of the signals carried at a given beat frequency and intensity using a Zeeman laser or a polarization generator. Firstly, the beauty of the two-frequency Zeeman modulation is to provide an orthogonal circular polarization states with an angular frequency difference, which can be used for a direct and convenience examination of the modulation to polarization sensitive FB couplers in SPPAM. Secondly, the polarization generator can be used for producing a period of polarization states with the tunable modulation frequencies, triggering the amplitude changes beyond the definite p-polarized mode at the dielectric/metal interface. In summary, in addition to miniaturization, our SPPAMs provide further merits of the relatively high polarization selectivity by the FB couplers^17^, noise-rejected ability by the two-frequency laser source^19^,^20^,^21^, and temporal stability to continuously encode the periods of polarization states^22^. The presence of the SPPAMs can be further explored for use in nanosensors^19^, plasmonic switches^23^, and other applications.

## Results

### Design and measurement of beat frequency SPPAM

**Figure 1:**
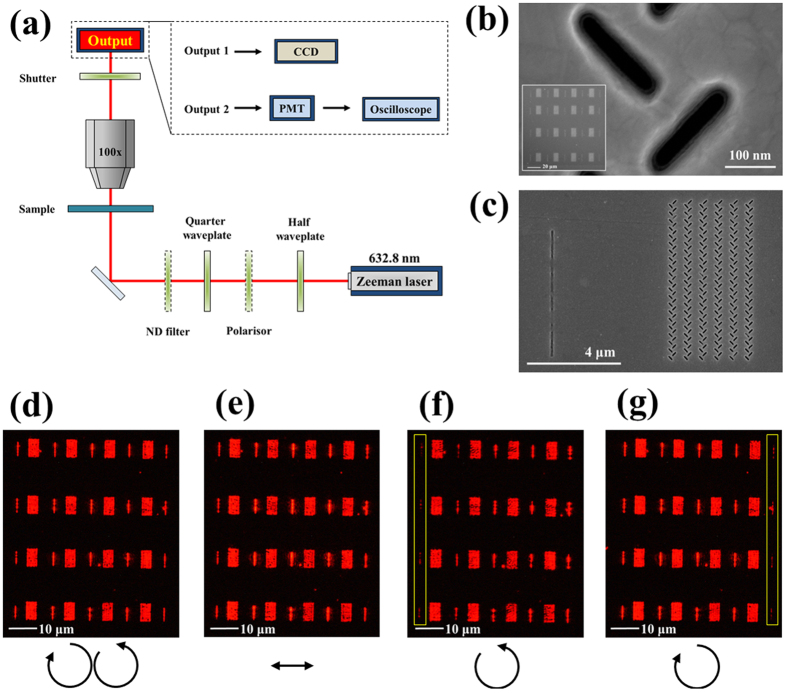
The setup of the beat frequency surface plasmon polariton amplitude modulation (SPPAM). (**a**) Optical setup for the measurement of the couplers. A light with the orthogonal linear polarized (LP) states passes through the wave plates (λ/2 and λ/4) and the polarizer, taking the phase delay and turn the polarization states into two circular polarized (R + LCP), LP, RCP, and LCP states. Furthermore, the nanofabricated Fishbone (FB) couplers are coupled by the polarized light from the free space couples and detected by the output paths (No. 1 is for CCD images and No. 2 is for the modulated waves). (**b**) SEM image of FB nanoarrays shows that the gaps of a single unit are 40 nm (Inset: low magnified 4 by 4 regions with the output slits). Note the output slits were not fully penetrated by the FIB milling process. (**c**) SEM image shows that a region of FB nanoarrays possesses 6 columns and 1 output slit, with a length and thickness of 5 μm and 100 nm, respectively. The CCD images of the 4 by 4 regions with the output slits show the excited couplers by different polarization states, including (**d**) R + LCP, (**e**) LP, (**f**) RCP, and (**g**) LCP states. Note the yellow squares show the contrast between the right and left side output slits.

The SPPAMs comprised the input light source, modulated controller, and SPP coupler to employ a channel, apply the signal, and excite the SPP. We used the experiments to demonstrate the selectivity of the directional coupling and the setup of producing the beat frequency (the angular-frequency difference) of the SPPAM, as shown in Fig. 1. First, we excited the SPP using a Zeeman laser. By applying a magnetic field in the He-Ne laser cavity, the associated Zeeman effect triggered the energy splitting due to two different electron spins, which led to two orthogonal lasing states of linear polarized (CP) light with an beat frequency of 1.66 MHz^19^. We then employed a half waveplate, a quarter waveplate, and a polarizer to control the distinct polarization states along the light path, giving rise to right + left circular polarized (R + LCP) lights, LP lights, RCP, and LCP lights, as shown in Fig. 1(a). The controlled polarized lights were transmitted through an array of 4 × 4 FB couplers (Fig. 1(b,c)), exciting SPP propagation along the air/Ag interface. The couplers were carefully tuned to the geometry factors, including the size of the unit slits (40.8 nm) and the distances between columns (611.7 nm) to match the dispersion relation to the air/Ag specification. An output slit in Fig. 1(c) couples the SPP to the far field with a length of 5 μm and a width of 100 nm. The SPP propagation length reached several micrometers, which indicated that the intensity is sufficiently strong to observe the signals. Finally, in accordance with the distinct polarization states of the excited lights, the SPP interfered with each other quite differently, and the interfered signal was extracted by a slit out-coupler with a thickness of 100 nm at the far field, as visualized by a CCD camera (i.e., output 1 detection path) and shown in Fig. 1(d–g).

**Figure 2:**
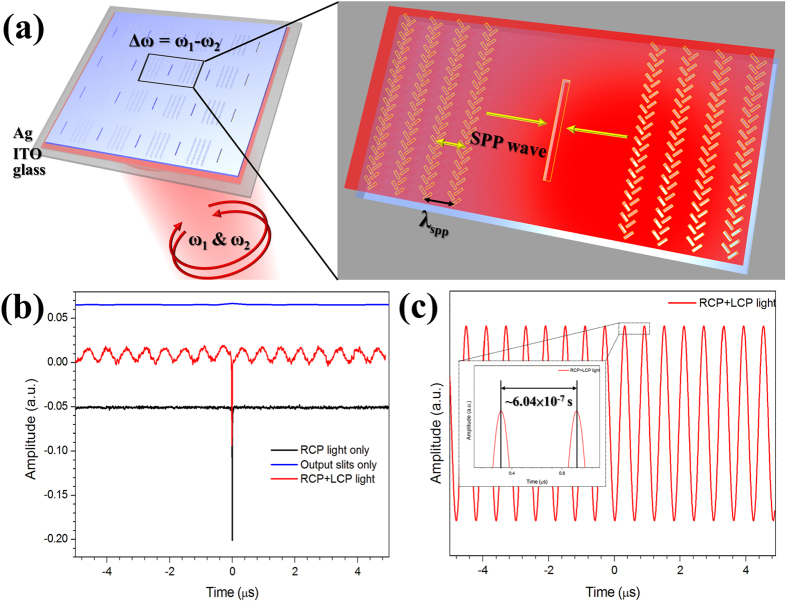
The modulated signals of beat frequency (angular frequency difference) SPPAM. (**a**) Scheme shows that the 4 by 4 regions of FB couplers with the outputs slits are fabricated on the Ag-coated ITO glass substrate. The CP lights with the angular frequencies (ω_1_ and ω_2_) are carried out for coupling on the sample, showing that the SPP waves propagate at the Air/Ag interface and interfere with the FB couplers (Yellow arrows). The modulated signals (beat frequency: Δω = ω_1_ − ω_2_) can be found from the both FB couplers and the output slits. (**b**) The modulated signals (Red curve) are readout by the oscilloscope as the comparisons of the RCP light coupled only (Black curve) and the output slit coupled only (Blue curve). (**c**) Fast Fourier transformed (FFT) filter is carried out for purifying the modulated signal, showing that the beat frequency is 1.66 MHz (1/6.04 × 10^−7^ s).

In fact, polarization states control the propagation directions of FB nanoarrays that determine the propagation directions to the right or left sides. In this case, the input of LP states can provide both the right- and left-side propagation (Fig. 1 (e)). Furthermore, to produce the maximum values of the extinction ratio, the input CP states play a vital role in matching the phase terms with the constructive and destructive interference between two channels (square slits with yellow color in Fig. 1(f,g)), which leads to unidirectional SPP propagation (see Supplementary Note 1)^17^. Therefore, on the nanofabricated FB nanoarrays, we employed a Zeeman laser that carries R + LCP states with an beat frequency to interfere with each other (see Fig. 2(a)), realizing a modulation frequency of 1.66 MHz, as shown in Fig. 2(b). Beside the modulated signal, we also measure two controlled groups by sending the RCP light with a RCP light only (black line) and by avoiding FB couplers (blue), respectively. Clearly, there appear no modulations in these two controlled measurements (see Supplementary Note 2). To manifest the measured modulated signal, we processed the raw data by the method of fast Fourier transform (FFT). After rejecting the noise and preserving the beating signals, there appears an unambiguous modulation signal of 1.66 MHz, as shown in Fig. 2 (c). Note that this modulation achieved by the beat frequency is stable and extremely sensitive^19^, and can be readily employed as an ultra-sensitive refractive index sensor^20^,^24^,^25^.

### Demonstration of polarization-controlled SPPAM

**Figure 3:**
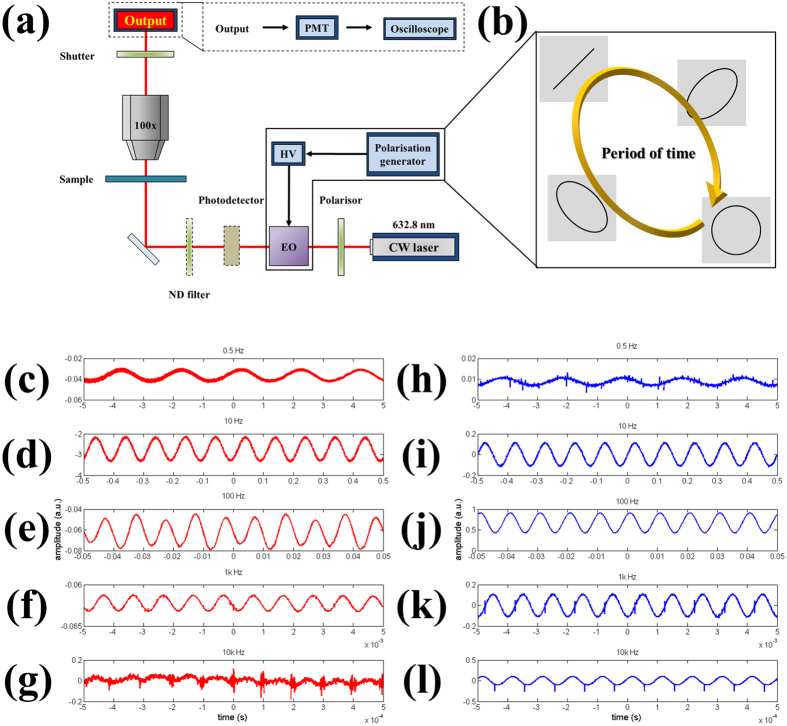
Optical setup and measurement of polarization-controlled SPPAM. (**a**) The design of the setup is similar to the beat frequency SPPAM. Notably, continuous wave (CW) laser and the polarization system (The polarization generator, High voltage component, and electro-optics (EO) crystal) are employed for controlling the polarization modulated signals and exciting the SPP waves on the sample. (**b**) The input modulated signals for the polarization-controlled SPPAM, showing that the polarization system can repeated change the polarization states in a period of time from a CP, ellicipity polarized (EP), LP, another EP, and back to CP states. The polarization-controlled modulated signals are observed by the oscilloscope. The control group measured by photodiode is employed for confirming that the status of input modulated signals, including (**c**) 0.5 Hz, (**d**) 10 Hz, (**e**) 100 Hz, (**f**) 1 kHz, and (**g**) 10 kHz. After that, the polarization-controlled SPPAM shows the results measured by the photomultiplier (PMT) in (**h**) 0.5 Hz, (**i**) 10 Hz, (**j**) 100 Hz, (**k**) 1 kHz, and (**l**) 10 kHz without observing the shifts and distortion.

In addition to applying the beat frequency, the SPPAM can be also realized by the polarization control. The experimental setup is illustrated in Fig. 3(a). Here, we replaced the Zeeman laser by a CW He-Ne laser as an input source, and integrated a polarization generator to provide the periodic polarization states. The polarization generator consists of a function generator, a high-voltage compressor, and an electro-optics modulator (EOM). Note that this polarization generator can select the polarization state and the period to modulate the light passing through the EOM, such that the intensity of the input signals varied with the cyclical polarization state, as presented in Fig. 3(b). Then, the modulated signals were projected on the FB couplers, exciting the SPP propagation. Since the propagation direction of SPP waves is determined by the polarization states on the FB couplers, the output slits on their right and left sides reveal a periodic change of signals (see Supplementary Movie and Fig. S2). Such modulated signals are monitored in free space, as displayed in Fig. 3(c–g). Furthermore, the modulated signals can be also experimentally demonstrated on the miniature chip by means of our SPPAM. It is evident to observe the modulated signals by the polarization-controlled SPPAM at frequencies of 0.5, 10, 100, 1 k, and 10 kHz, as shown in Fig. 3(h–l), which indicate no signal distortion or peak shifting compared with modulation in the free-space propagation.

### Extinction ratios of left- and right-side SPP waves by encoding a Poincaré sphere

**Figure 4:**
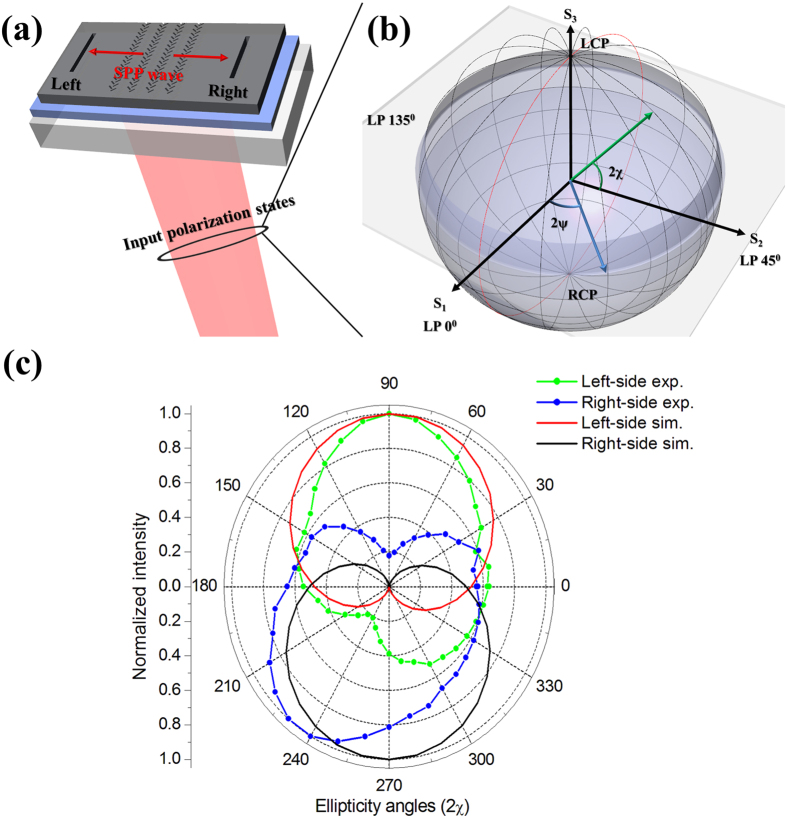
Extinction ratio of SPPAM. (**a**) The designed scheme shows the simulation and the measurement methods for the extinction ratios of SPP waves between the left and the right sides. (**b**) Poincaré sphere is used for systematically encoding the input polarization states. Note that the azimuth and ellipticity angles are denoted with 2ψ and 2χ. Herein, the azimuth angle is fixed at 0^o^ and the ellipticity angles are swept for the simulation and the measurement results in (**c**). The trajectory for the sweeping is marked with the red loop. (**c**) The polar diagram shows the simulation and measurement results between the left and the right sides of the output slits, noted that the intensities are normalized by means of |E|^2^/|E_max_|^2^.

By means of both the beat frequency from the Zeeman splitting and polarization control from the polarization generator, we demonstrated two types of SPPAMs. The common enabling factors for the SPPAMs are the selection of SPP propagation direction and the quality of the controlled polarization states. To scrutinize these two factors, it is demanded to determine the extinction ratio between the right and left propagation directions of the SPPAM. The configuration of the SPPAM is presented in Fig. 4(a), in which the distance between the FB couplers and the right/left outputs is the same, for a fair comparison. The carried polarization states in the input light source were systematically followed by a sweep of a Poincaré sphere to explore each point on a sphere formed by the variation of the azimuth angle (2ψ) and ellipticity angle (2χ), as previously shown in Fig. 3(b) 26. Here, we denote three axes of S_1_, S_2_, and S_3_ as 0° polarized LP, 45° polarized LP, and LCP, respectively. The amplitudes are fixed such that all states are located on the surface of the Poincaré sphere. A circle looped along the S_1_ and S_3_ axes is marked with a red line to represent the direction from RCP, 0° LP, LCP, and back to RCP, and the azimuth angle was fixed at 0°, and the ellipticity angle was swept in the 0–360° range, as shown Fig. 4(b). The propagated SPP waves were numerically calculated by the finite-difference time domain (FDTD) and experimentally collected by output slits. Actually, the function of the FB coupler is to direct the SPP wave, which can be evaluated by the normalized extinction ratio between the right and left outputs at the same polarization states^13^ 
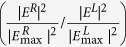
. The results of the normalized intensity with the ellipticity are shown in Fig. 4(c). The simulated maximum extinction values between the right and left output slits indeed appear at 90° and 270° by the incidence of LCP and RCP states, and the experimental measurement also indicates the similar result in the range of ellipticity angles 90° (LCP) and 230–240° (RCP), respectively.

**Figure 5:**
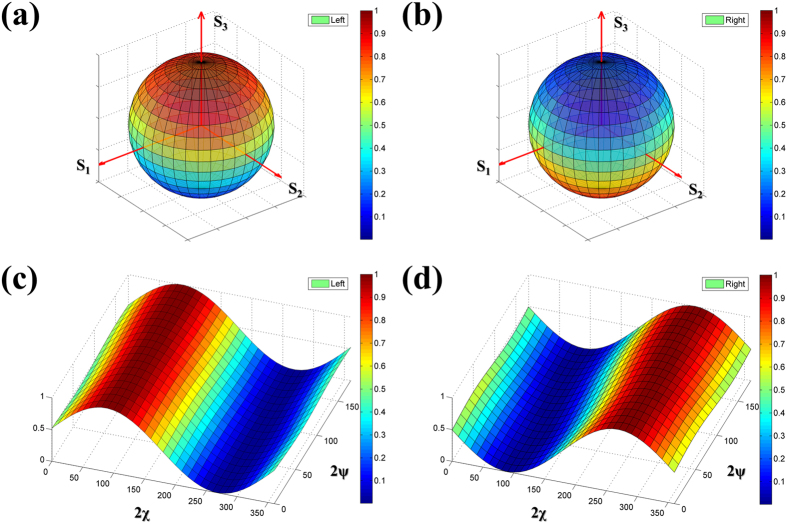
2D mapping for the extinction ratios of SPPAM. (**a**) Simulation results for left side SPP waves and (**b**) the right side SPP waves are shown by systematically encoding the input polarization states with a Poincaré sphere. (**c**,**d**) are the normalized E-field intensities between the ellipticity angle (2ψ) and azimuth angle (2χ), showing that the detailed plots in the ranges of 0° ≤ 2χ ≤ 360° and 0° ≤ 2ψ ≤ 180°. The contrast between the left and right side simulations indicates the relationship between the input polarization states and the output SPP intensities.

To further manifest the polarization control, we mapped 2D diagrams of sweeping the entire Poincaré sphere by controlling the azimuth and ellipticity angles. Fig. 5(a,b) demonstrate the composition of the spheres and show that the resolution of one pixel is limited to the 10 × 10 spacing. The normalized intensities at the same spatial positions were carried out for the comparable color changes with the 0–180° range of 2ψ and the 0–360° range of 2χ. The curved meshes, colored to visualize the normalized intensity, are the simulation results for the 1D scanning processes along both 2ψ and 2χ. The spheres shown in Fig. 5(a,b) refer to the input polarization states that excite the FB couplers and the output intensities in the left and right directions. Obviously, the intensity of out-coupling signals is opposite at the north and south poles. Furthermore, Fig. 5(c,d) detail the plot of the polarization-controlled output intensities. When the ellipticity angles are fixed in the ranges of 90–100° and 270–280°, these plots present that the normalized intensities approximate 1 (maximum) and 0.01 (minimum) between the left and right simulations. On the other hand, the contrast between the left and right simulation is revealed when the azimuth angles are fixed, indicating that the SPPAM can be also manipulated by the latitude of Poincaré sphere. More importantly, each of polarization states gives a potential of the code number for the modulation signals, depending on the frequencies of varying polarization states by the polarization generator and the separations of identifying output intensities by the CCD system. We believe that the propagation of SPP waves can be well directed by polarization states in the SPPAMs.

## Discussion

Unlike the basic Mach-Zehnder (MZ) interferometer construction^4^, compact SPP modulators reduce the required power dissipation and long light path in the optical setup. The propagating SPP waves at the air/Ag surface possess a non-aligned and on-chip solution for the miniaturization of the compact constructions. In order to direct the SPP waves propagation, FB couplers with the output slits play a vital role in the transmission of modulated signals because of the polarization-controlled SPP directional propagation. Using the normal incident excitation, we only need to control the polarization states instead of making additional efforts to manipulate both the polarization and the incident angle for mode matching^15^. The collective coupling among FB arrays enhances the switchable SPP propagation, directing the output intensities at the couplers^17^. This is suitable to be a core controller for the SPPAM.

We provide two types of SPPAM based on the FB couplers and envision that our approach can be explored to actuators^27^ and optical choppers^28^. First, the beat frequency SPPAM can preserve the 1.66 MHz AC terms when the fluctuation occurs in the spatial domain. This is a possible route for stabilizing two frequency signals and coupling the beat frequency for use as a sensor^19^,^20^,^24^,^25^. Second, polarized-controlled SPPAM preserves the original period frequency along the Poincaré sphere. The signals in the output slits may act as a functional polarized stabilizer that can be used to stabilized the intensities during the on-chip SPP nanodevice^29^ manipulation.

Furthermore, in regard to the signal processing, the constellation diagrams are usually employed to modulate and key the symbols. In the typical 8 or 16 coding, the digital signals are obtained by controlling the phase and amplitude modulations, as used in phase-shift keying (PSK)^30^ and quadrature amplitude modulation (QAM)^31^. In the polarized-controlled SPPAM, the modulated signals can be encoded with the polarization states such that the allowable symbols may increase beyond the sub-wavelength optical applications. In other words, both the periods of rotating frequencies of polarization states and the polarization states of the output intensities can function as the SPP modulation codes. The 2D mapping simulation presented in Fig. 5 shows the possibility of encoding with high selectivity and quality for the SPP propagation direction and the controlled polarization states. The results show that the values of high extinction ratios and conversion efficiencies in the simulations and the experiments under 10° angle rotation (see Supplementary Note 3). This would open a new route for compact SPP modulation.

## Conclusion

In conclusion, we demonstrated the miniature beat-frequency and polarization-controlled SPPAMs, modulating the signals with the Zeeman laser and the polarization generator, respectively. On one hand, the beat-frequency SPPAM is operated at an angular frequency difference of 1.66 MHz (RCP + LCP states); on the other hand, the polarization-controlled SPPAM is operated at a frequency ranging from 0.5–10 k Hz. In these two kinds of SPPAMs, the modulated signals are coupled and directed by the nanofabricated FB couplers. To manifest the extinction ratio of SPP waves by polarization control, we systematically steered the input polarizations on the Poincaré sphere, and observed an excellent normalized extinction ratio up to 100. Both the simulation and experiment are in a good agreement. These two new SPPAM methods can be readily used for multi-bit plasmonic modulators, ultrasensitive plasmonic nanosensors, and plasmonic nanocircuits in future studies.

## Methods

### Fabrication of Fishbone couplers

FB couplers were designed by Lin *et al*.^17^. Originally, the geometry factors of the coupler were investigated on an Au-coated substrate under 632.8-nm laser excitation. Here, we replaced the coating with the Ag-coated substrate and changed the geometry factors to S = 152.93 nm, D = 305.85 nm, W = 40.78 nm, L = 203.90 nm, and column-to-column space = 611.70 nm to compete with the real part of dielectric constant for Ag (Re(ɛ_Ag_)). A 150 nm Ag surface on Indium Tin Oxide (ITO, thickness: 5 nm) glass substrate was fabricated by using the E-gun evaporation process. The evaporation rate was 0.5 Å/sec. FB couplers were fabricated by using the focused ion beam (FIB, FEI Helios NanoLab 600i) milling process. In the milling process, the scale of FB couplers was followed by the geometry factors and the output slits were milled with a length of 5 μm and a width of 100 nm. Note that the depths of output slits were not be penetrated to the ITO layer so that the slits were not coupled by the transmitted laser light. The ion gun voltage and current in the milling process were fixed at 30 kV and 24 pA. The sample was then measured using the optical setup.

### Optical Measurement

Two optical setups for the SPPAMs were shown in Figs 1(a) and 3(a). Firstly, the setup in Fig. 1(a) was used to measure the polarization-controlled FB couplers and the beat frequency SPPAM. Zeeman laser (632.8 nm, Agilent Technologies, 5517A), half wave plate, polarizer, and quarter wave plate were used to tune the polarization states, guiding the signal into the optical microscope system (Nikon, 50i phase microscope), as observed by the 100x objective lens (Nikon, LU Plan Fluor BD). All beams from free space for the excitation of the couplers were normal incident. The output 1 path with the charge-coupled device (CCD) is to observe the images of FB couplers and the output slits. On the other hand, the output 2 path with the PMT (Zolix Instruments, PMTH-S1-CR131) and the oscilloscope (Agilent Technologies, DSO-X-2024A) is to measure the AC signals from the SPPAMs in the average or high-resolution mode. Secondly, a CW laser source (He-Ne laser, 632.8 nm, Newport, R-32734) was used to observe the extinction ratio and the polarization-controlled SPPAM. The extinction ratios were calculated from a series of CCD captured images, noted that the captured images were fixed the working distances and the exposure time to interpret the left- and right-side intensities from the output slits at the same distances between the FB couplers and the output slits. Furthermore, using the software (Nikon, DS-Fi2 system), the intensities were normalized by the maximum values of |E_max_|^2^ and plotted together with the simulation results at the azimuth angle (2ψ) = 0^o^. The polarization-controlled SPPAM signals were also measured by using the PMT and the oscilloscope, which was carried out for measuring the modulated signals from the polarization generator and read out the periods of the polarized frequencies from 0.5 Hz to 10 k Hz.

### FDTD simulation

Lumerical FDTD solution was used for calculating the SPP wave propagations of FB couplers. To systematically control the incidence of polarizations, we employed a Poincaré sphere and denoted the principle axes S_1_, S_2_, and S_3_ with the Azimuth angle (2ψ, [0^o^ 180^o^]) and the ellipticity (2χ, [0^o^ 360^o^]). The FB couplers were illuminated by 632.8 nm Gaussian beam with varying the 2ψ and 2χ to go through the Poincaré sphere. Perfectly matched layer (PML) was carefully used as the boundary condition in Cartesian coordinate and the materials database was followed by Palik (0–2 μm). We also applied the 5-nm ITO layer between the 120-nm Ag layer and the glass substrate. Two monitors were used to recode the coupled E-field intensities from the right and left sides, and the points were plotted for the normalized intensity (|E|^2^/|E_max_|^2^) curves from the left and right sides. The distance between the monitors and the FB structures was 5 μm, and the distances for the left and right monitors were equal.

## Additional Information

**How to cite this article**: Chang, C.-W. *et al*. Miniature Surface Plasmon Polariton Amplitude Modulator by Beat Frequency and Polarization Control. *Sci. Rep.* **6**, 32098; doi: 10.1038/srep32098 (2016).

## Supplementary Material

Supplementary Information

Supplementary video
