# Supplementary material for: Miniature Surface Plasmon Polariton Amplitude Modulator by Beat Frequency and Polarization Control
Source: Sci Rep. 2016 Aug 25;6:32098. doi: 10.1038/srep32098 (PMC4997335; doi:10.1038/srep32098)
Supplement: Supplementary Information [file srep32098-s1.docx]

**Supplementary Information**

**Miniature Surface Plasmon Polariton Amplitude Modulator by Beat Frequency and Polarization Control**

**Cheng-Wei Chang^1, +^, Chu-En Lin^2, +^, Chih-Jen Yu^3^, Ting-Tso Yeh^1^, and Ta-Jen Yen^1, 4, *^**

^1^Department of Materials Science and Engineering, National Tsing Hua University, 101, Section 2, Kuang Fu Road, Hsinchu 30013, Taiwan

^2^Department of Mechanical Engineering, National Chin-Yi University of Technology, Taichung 41170, Taiwan

^3^Graduate Institute of Electro-Optical Engineering, Chang Gung University, Taoyuan 333, Taiwan

^4^Center for Nanotechnology, Materials Science, and Microsystems, National Tsing Hua University

^*^[tjyen@mx.nthu.edu.tw](mailto:tjyen@mx.nthu.edu.tw)

^+^These two authors contribute to this work equally.

**Supplementary Note 1. The beat frequency (1.66 MHz) detected by the PMT under Zeeman laser excitation**

Following the Ref. 17, we assume that the waves are monochromatic, time is independent to the spatial domain, and the propagated waves are plane wave. Therefore, the Zeeman laser source generates two propagation waves with the orthogonal linear polarized (LP) states.

$E_{1}=E_{1}^{0}\exp i(-kr_{1}+\omega_{1}t+\delta_{1})$ (1)

$E_{2}=E_{2}^{0}\exp i(-kr_{2}+\omega_{2}t+\delta_{2})$ (2)

, where *E*_1_ and *E*_2_, *k*, Δ*ω* = *ω*_1_ - *ω*_2_, and *δ*_1_ and *δ*_2_ are the two incident E fields, wavevector, the Zeeman-splitted angular frequency difference (beat frequency), and the initial phase terms (Δ*δ* = *δ*_1_-*δ*_2_). Note that the *r*_1_ and *r*_2_ are used for serving as the distance between the semi-columns of the Fishbone (FB) array.

Let

$A_{1}=-kr_{1}+\omega_{1}t+\delta_{1}$ (3)

$A_{2}=-kr_{2}+\omega_{2}t+\delta_{2}$ (4)

Intensity of two semi-columns (*I*_2_) under Zeeman laser excitation can be expressed as

$I_{2}=\left| E_{1}+E_{2} \right|^{2}$=$\left| E_{1}^{0}exp(iA_{1})+E_{2}^{0}exp(iA_{2}) \right|^{2}={|E_{1}^{0}\cos A_{1}+iE_{1}^{0}\sin A_{1}+E_{2}^{0}\cos A_{2}+iE_{2}^{0}s{\mathrm{in}A}_{2}|}^{2}$ (5)

$\to I_{2}={(E_{1}^{0}\cos A_{1}+E_{2}^{0}\cos A_{2})}^{2}+{(E_{1}^{0}\sin A_{1}+E_{2}^{0}s{\mathrm{in}A}_{2})}^{2}={(E_{1}^{0})}^{2}\cos^{2}A_{1}+{(E_{2}^{0})}^{2}\cos^{2}A_{2}+{(E_{1}^{0})}^{2}\sin^{2}A_{1}+{(E_{2}^{0})}^{2}\sin^{2}A_{2}+2E_{1}^{0}E_{2}^{0}\sin A_{1}\sin A_{2}+2E_{1}^{0}E_{2}^{0}\cos A_{1}\cos A_{2}$ (6)

If the amplitude is $E_{1}^{0}=E_{2}^{0}=E$ and ${{(E}_{1}^{0})}^{2}={{(E}_{2}^{0})}^{2}=2E^{2}$; then we obtain

$I_{2}=2E^{2}+2E^{2}\cos\left( A_{1}-A_{2} \right)=2E^{2}+2E^{2}\cos\left[ -k\left( r_{1}-r_{2} \right)+\left( \omega_{1}-\omega_{2} \right)t+\left( \delta_{1}-\delta_{2} \right) \right]=2E^{2}+2E^{2}\cos\left( -kS+\Delta\omega t+\Delta\delta\right)$ (7)

Note that the semi-column distance between *r*_1_ and *r*_2_, where *S* = *r*_1_ - *r*_2_.

- 1. Circular polarized (CP) light without the angular frequency difference:

The phase term Δ*δ*=$\frac{\pi}{2}$, when the propagation waves are orthogonal.

$k=k_{\mathrm{spp}}=\frac{2\pi}{\lambda_{\mathrm{spp}}}, S=\frac{\pi}{2k_{\mathrm{spp}}}, and kS=\frac{\pi}{2}$

We get

$I_{2 Right}=2E^{2}+2E^{2}\cos\left( -kS+\Delta\delta\right)=4E^{2}$ (8) $I_{2 Left}=2E^{2}+2E^{2}\cos\left( -kS-\Delta\delta\right)=0$ (9)

The result is similar to Ref. 18 and confirms that the FB couplers can direct the SPP propagation directions. The maximum extinction ratio between right- and left-ended sides (*I*_2 Right_ and *I*_2 Left_) is under CP light excitation.

- 1. CP light with considering the beat frequency:

$I_{2}=2E^{2}+2E^{2}\cos\left( -kS+\Delta\omega t+\Delta\delta\right)=2E^{2}+2E^{2}cos(\Delta\omega t)$ (10)

Eqn. (10) reveals two terms of the interfered intensity, the fixed (or DC) term $2E^{2}$ and time-varying (or AC) term $2E^{2}cos\Delta\omega t$. Such a result indicates that the $\Delta\omega$ (i.e. the beat frequency split from the Zeeman laser) eventually contributes to amplitude modulation.


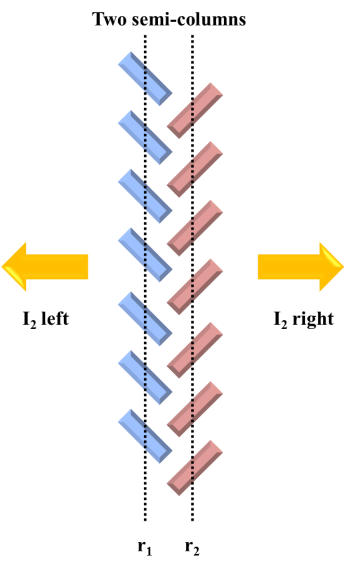


**Supplementary Figure S1|** **Schematic for the calculated FB couplers.** The figure shows that two semi-columns are located at the *r*_1_ and *r*_2_ directions with a distance *S* and the intensities of SPP propagation are marked with two directions (*I*_2_ _left_ and *I*_2_ _right_).

**Supplementary Note 2. A discussion of controlling the polarization states under Zeeman laser excitation**

The use of polarization states in the beat-frequency SPPAM are two orthogonal R+LCP states and one RCP state. In Fig. 2(b), only the R+LCP states reveal the beat frequency because the angular frequency difference is encoded that R+LCP states are the ω_1_ and ω_2_. Note that the beating process also happens with employing two orthogonal R+LCP states since a CP state can be decomposed to two LP states. 2 groups of orthogonal LP states with the angular frequency difference are carried out for the modulation and thus beat on the FB couplers. On the other hands, the RCP state was produced by carefully matching one of the orthogonal LP states and allowed the light source when passing through a quarter waveplate as shown in Fig. 1(a). Therefore, only an angular frequency was carried by the light source without the beating frequency (Δω).

**Supplementary Movie. Movie for the polarization-controlled SPPAM.**


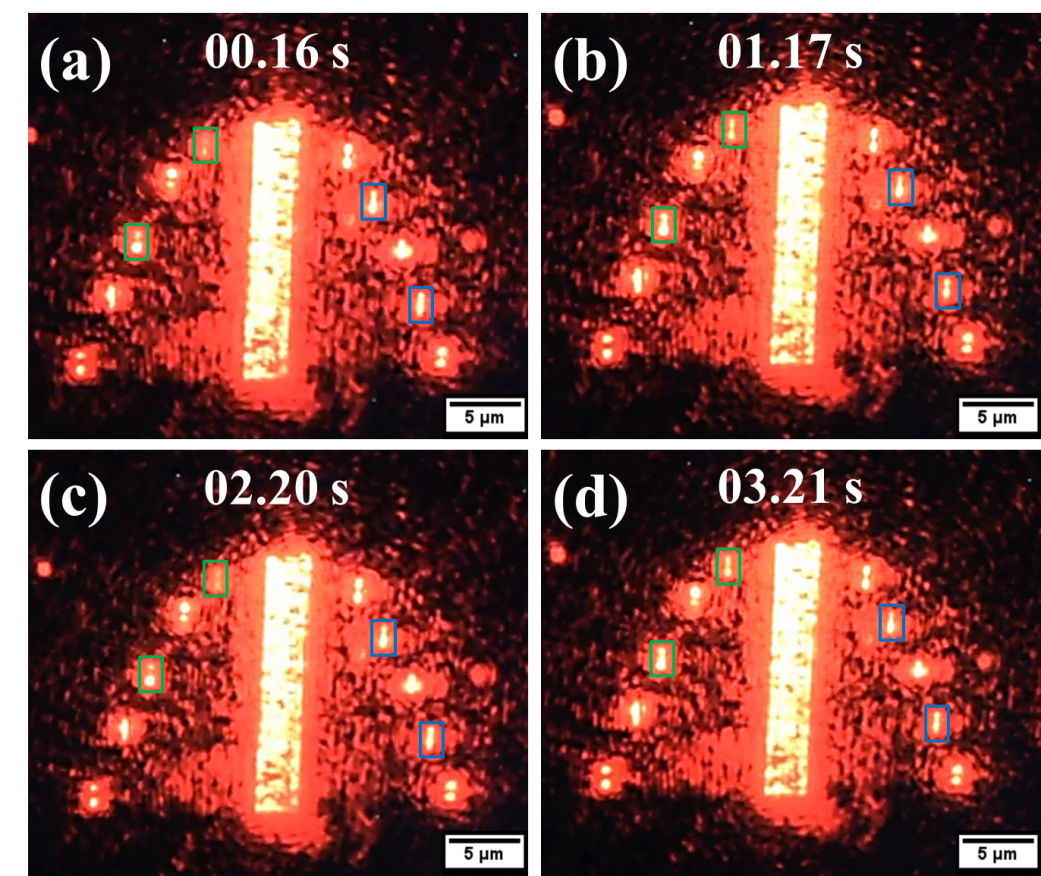


**Supplementary Figure S2 | OM images of the FB couplers modulated by the polarization system.** The coupled lights encoded with the modulated signals show that intensities change at the different periods of time (**(a)** 0.16 s, **(b)** 1.17 s, **(c)** 2.20 s, and **(d)** 3.21 s), which nearly equal to the input of the modulated frequency (1 Hz). Note that the marked squares are the contrast between the left and the right side output slits and the images are captured from the movie for the polarization-controlled SPPAM.

**Supplementary Movie Legend**

**Supplementary Movie 1 | polarization-controlled SPPAM at 1 Hz modulation frequency.** The modulated light coupled with the FB couplers arrays at the center, showing the intensities change between the right/left side output slits. Note that the images were captured and shown in Fig. S2 (a)-(d).

**Supplementary Note 3. A discussion of the loss in SPPAMs**

In the SPPAM demonstrations, the readout of left/right output silts dominates the identification of the modulation signals. This is because that the FB couplers direct the SPP wave propagation, which encodes the beat frequency and the rotating polarization frequencies to the observation. Therefore, Fig. 4(c) and Fig. 5(c)-(d) represent the relationships between the input polarization states and the output intensities. The simulation results give the maximum extinction ratio at the value of 1/0.01=100 (Right-to-left side and Left-to-right side) in the CP states; the experiments with a consideration of the free-space coupling efficiency show the given value of 0.81/0.39=2.07 (Right-to-left side) and 1/0.17=5.88 (Left-to-right side) in the maximum CP states. The results are in the agreement that FB couplers can identify the intensity difference with the encoded modulation frequencies. Under the CP states excitation, FB couplers can only allow one-sided channel propagation, so the principally the other side is closed by the subtractive SPP waves. The optimized conversion efficiencies^28^ ($\frac{\frac{|E^{L}|^{2}}{|E_{\max}^{L}|^{2}}-\frac{|E^{R}|^{2}}{|E_{\max}^{R}|^{2}}}{\frac{|E^{L}|^{2}}{|E_{\max}^{L}|^{2}}+\frac{|E^{R}|^{2}}{|E_{\max}^{R}|^{2}}}$) are 98.02% in simulation and 70.94% in experiment, respectively. In addition, the nature quality of the Ag thin film is another crucial issue to the SPP wave propagation. Such the surface defects, surface scattering, and band transitions lead to the propagation loss^12,29^. In our previous report, the single crystalline Ag microplates^34^ have a potential to tackle the problem. Since the FB coupler is very sensitive to the polarized light, we herein have not carry out the objective lens (OL) to focus the input laser. Nevertheless, the scarification of the small spot size is worthy and can prevent the polarization states from the distortion under the high magnification OL focusing.
